# Phosphatidylinositol 3-kinase-δ (PI3K-δ) is a potential therapeutic target in adult T-cell leukemia-lymphoma

**DOI:** 10.1186/s40364-018-0138-7

**Published:** 2018-07-18

**Authors:** Hiroo Katsuya, Lucy B. M. Cook, Aileen G. Rowan, Yorifumi Satou, Graham P. Taylor, Charles R. M. Bangham

**Affiliations:** 1 Section of Virology, Department of Medicine, Imperial College London, W2 1PG, London, UK; 2 Centre for AIDS Research, Kumamoto University, Kumamoto, Japan; 3 International Research Center for Medical Sciences, Kumamoto University, Kumamoto, Japan

**Keywords:** Adult T-cell leukemia-lymphoma, HTLV-1, PI3k-δ, Idelalisib

## Abstract

The prognosis of adult T-cell leukemia-lymphoma (ATL) remains very poor, and there is an urgent clinical need to investigate novel therapies for ATL. The expression of phosphatidylinositol 3-kinase-δ (PI3k-δ) is normally restricted to hematopoietic cells and is known as a key determinant of cell survival in certain cancers. The inhibitor of PI3k-δ, idelalisib, has been shown to be effective in the treatment of chronic lymphocytic leukemia. Here, we report the expression of PI3k-δ and the ability of idelalisib to promote apoptosis in ex vivo ATL samples. The activity of PI3K was quantified by a PI3-Kinase Activity ELISA kit. Although there was no significant difference in mean PI3K activity between healthy donors and patients with ATL, certain cases of ATL showed extremely high PI3K activities. The expression of PI3k-δ protein was detectable in most ATL cases. The freshly isolated cells from ATL patients were cultured with or without idelalisib for 0–10 days, and cell survival was then quantified. Idelalisib induced apoptosis in ATL cells in a time-dependent manner, and significantly reduced the frequency of viable ATL cells at 10 days. No time-dependent effects of idelalisib were observed in non-malignant T cells from the same patients. CCL22 has been reported to promote survival of ATL cells in part through the PI3K-AKT pathway. Idelalisib blocked this CCL22-induced phosphorylation of AKT and significantly inhibited the proliferation of ATL cells. These results validate the PI3K-AKT pathway as a potential therapeutic target in ATL.

**Electronic supplementary material:**

The online version of this article (10.1186/s40364-018-0138-7) contains supplementary material, which is available to authorized users.

## Main text

Adult T-cell leukemia-lymphoma (ATL) is a malignancy of peripheral T lymphocytes caused by infection with human T-lymphotropic virus type-I (HTLV-1). Approximately 5% of HTLV-1 carriers develop ATL in their lifetime [1]. ATL is classified into 4 clinical subtypes based on clinical features; acute, lymphoma, chronic and smoldering subtypes [2]. The chronic type is further divided into favorable and unfavorable chronic types, using objective criteria. The acute, lymphoma, and unfavorable chronic types are collectively classified as aggressive ATL. At present, first-line treatment for patients with aggressive ATL consists of chemotherapy using multi-cytotoxic agents together with a humanized anti-CCR4 monoclonal antibody or anti-retroviral therapy (i.e. with interferon-α plus zidovudine) [3]. The prognosis of aggressive ATL remains very poor [4] and there is an urgent clinical need to investigate novel therapies for ATL.

The phosphatidylinositol 3-kinase (PI3K) pathway, a key signaling system that links multiple receptors and oncogenes, is commonly activated in human cancers. PI3K-δ regulates the function of cells in the immune system: its expression is mostly restricted to hematopoietic cells, including myeloid cells, B cells, and T cells. In mouse models, inactivated PI3K-δ impaired antigen receptor signaling in B and T cells, and attenuated the immune response [5].

The PI3K-δ inhibitor, idelalisib, significantly improved survival in patients with relapsed chronic lymphocytic leukemia (CLL) with a tolerable safety profile [5]. PI3K-δ also plays an important role in the normal functions of several T-cell subsets [6]. In ATL, activation of the PI3K pathway was found to be associated with formation of the multilobulated nucleus, and cell proliferation [7]. In cells transfected with the gene encoding the HTLV-1 trans-activator protein and oncoprotein Tax, phosphorylated AKT was upregulated, through down-regulation of PTEN (phosphatase and tensin homolog) [8]. In addition, mutation of the chemokine receptor CCR4, which is expressed on ATL cells and is a promising therapeutic target in ATL, enhanced the PI3K/AKT pathway after receptor engagement by C-C motif chemokine 22 (CCL22) [9]. These findings indicate that PI3K-δ is a potential therapeutic target in ATL. Here, we demonstrate that idelalisib induces apoptosis and cell toxicity in ATL cells in vitro, and overcomes stimulation by a cytokine, CCL22.

**Fig. 1:**
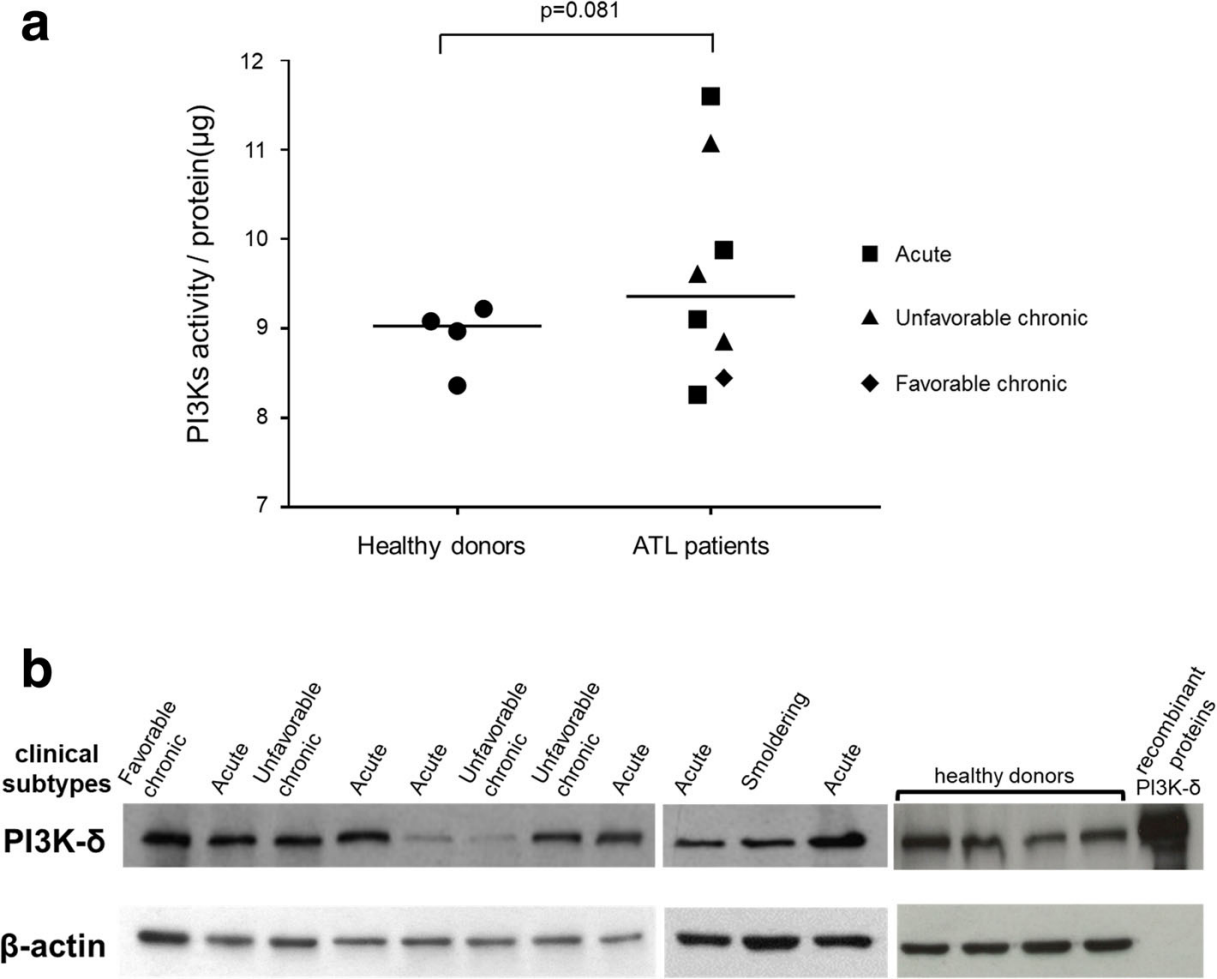
PI3K activity and expression of PI3K-δ in ATL cells. **a** The activity of PI3K was quantified by PI3-Kinase Activity ELISA kit (Echelon). Whole-cell lysates were extracted from isolated CD4^+^ cells using Dynabeads (Invitrogen) from the PBMCs of healthy donors (*n* = 4) and ATL patients (*n* = 8). **b** PI3K-δ expression was identified by immunoblotting using the PBMCs of HTLV-1 uninfected healthy donors (*n* = 4), and ATL patients in whom the ATL clone constituted more than 75% of PBMCs (*n* = 11)

The PI3K activity assay was performed on whole-cell lysates from ATL patients and HTLV-1 uninfected donors. Although there was no significant difference in mean PI3K activity between healthy donors and patients with ATL, certain cases of ATL showed extremely high PI3K activities (Fig. 1a). The level of PI3K activity varied across all ATL subtypes, and there was no clear relationship to the subtype or prognosis. The expression of PI3k-δ protein was subsequently assayed by western blotting of peripheral blood mononuclear cells (PBMCs) from 11 patients, in each of whom the ATL clone constituted over 75% of, and from 4 healthy donors (Fig. 1b). The expression of PI3k-δ varied between individuals with ATL, although it was detectable in most cases.

**Fig. 2:**
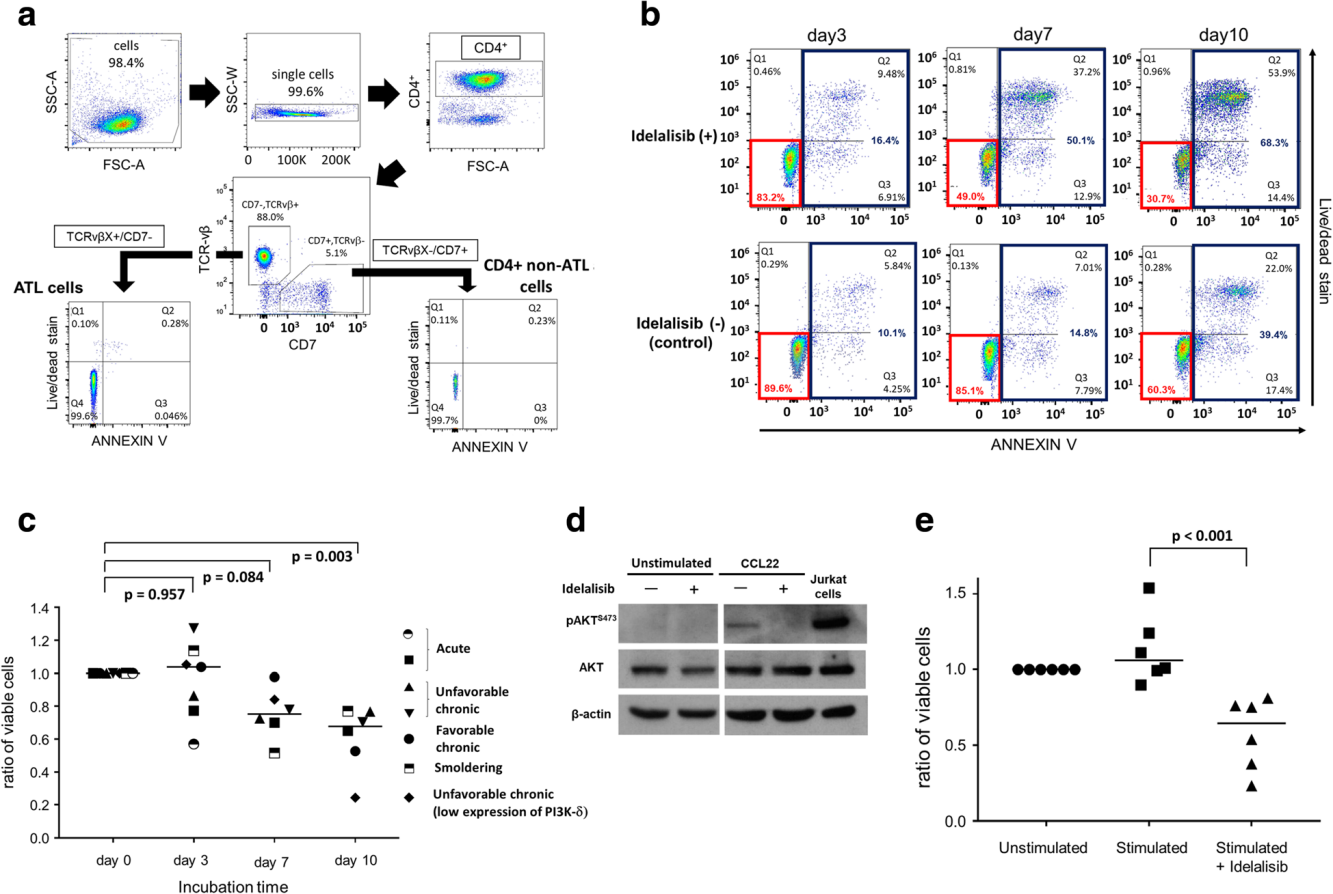
Effect of idelalisib on fresh primary ATL cells and non-malignant cells from ATL patients. **a** Flow-cytometry gating strategy. Uncultured PBMCs were screened using a panel of monoclonal antibodies (Beckman Coulter IOTest Beta mark) to identify the TCR-Vβ subunit expressed by the malignant clone, as previously described [13]. The viability of ATL cells was assayed after staining with annexin V (BioLegend) and Fixable Dead Cell Stain (Thermo Fisher). **b** Flow-cytometric assay in a patient with unfavorable chronic type. The gate with AnnexinV¯ and Live/Dead¯ stains, highlighted in red, indicates viable cells, and the gate with AnnexinV^+^, highlighted in blue, shows apoptotic cells. **c** The viability of ATL cells was assayed at day 0–10 by flow cytometry. PBMCs were freshly isolated from ATL patients (*n* = 7) and CD8 positive cells were depleted using Dynabeads (Invitrogen). The live and apoptotic cells were quantified in the population of TCR-VβX^+^CD7^−^ ATL cells. Y axis refers to the viability ratio between treated and untreated cells of time-matched samples from the same individuals. **d** CD4^+^ cells were selected from freshly isolated PBMCs and pre-incubated with or without idelalisib for 30 min. These cells were subsequently cultured with or without 50 μg/ml of CCL22 (R&D SYSTEMS) for 2 h and the whole cell lysates extracted for immunoblotting of AKT and AKT phosphorylated at Ser473. **e** Freshly isolated PBMCs from ATL patients (*n* = 6) were pre-incubated with or without idelalisib for 30 min, after depletion of CD8+ cells. These cells were subsequently cultured with or without 50 μg/ml of CCL22 for 0–10 days. All flow cytometric assays were performed in duplicate

We investigated the effect of idelalisib on the viability of ATL cells in vitro. First, frozen and thawed PBMCs of four ATL patients were incubated with or without idelalisib (0.1-100 μM) for 72 h. This concentration range was selected on the basis of prior reports of preclinical activity of idelalisib in CLL [10]. Samples from three of the four patients showed a dose-dependent decrease in cell viability (Additional file 1: Figure S1). We subsequently tested the effect of idelalisib in ATL using freshly isolated cells, to avoid the possible confounding effect of spontaneous cell death in cryopreserved cells. PBMCs were isolated from 7 patients: 2 patients with acute type, 2 unfavorable chronic type, 1 favorable chronic, 1 smoldering type, and 1 unfavorable chronic type with low expression of PI3K-δ by the immunoblotting assay. The freshly isolated cells were cultured with or without 10 μM idelalisib for 0–10 days. ATL cells were discriminated from non-ATL cells by flow cytometric staining for the TCR-Vβ subunit and CD7 (Fig. 2a). A representative example of the resulting flow-cytometric analysis is shown in Fig. 2b. Idelalisib induced apoptosis in ATL cells in a time-dependent manner, and significantly reduced the frequency of viable ATL cells at 10 days even in the sample with low expression of PI3K-δ (Fig. 2c, Additional file 1: Figure S2). No time-dependent effects of idelalisib were observed in non-malignant T cells from the same patients (Additional file 1: Figures S3, S4).

CCL22 binding to CCR4 has been reported to promote survival of ATL cells, in part through the PI3K-AKT pathway [9]. Exposure of primary ATL cells to CCL22 induced phosphorylation of AKT at Ser473. Idelalisib blocked this CCL22-induced phosphorylation of AKT (Fig. 2d). Furthermore, idelalisib significantly inhibited the proliferation of ATL cells in the presence of CCL22 (Fig. 2e).

The findings reported here closely resemble the impact of idelalisib on ex vivo cells from patients with CLL, in which idelalisib has a potent therapeutic benefit [5, 10]. Despite this clinical benefit in CLL, neither PI3K-related mutations nor alteration of PI3K expression have been observed in CLL patients. However, inactivating mutations and deletion of PTEN have been observed in CLL [11]. Idelalisib also showed an effect even on ATL cells with low expression of PI3K-δ. The alteration of PTEN may be associated with a favorable response to treatment with PI3K inhibitors. Furthermore, idelalisib is known to be active in cases of CLL with TP53 mutation or deletion, which are also frequently observed in aggressive ATL [12]. These results validate the PI3K-AKT pathway as a potential therapeutic target in ATL.

In conclusion, idelalisib significantly reduced the survival of primary ATL cells ex vivo and blocked CCL22-induced phosphorylated AKT. As in CLL, idelalisib monotherapy is unlikely to be sufficient to treat ATL, but these data support the investigation of idelalisib as part of combination therapy or as maintenance therapy for ATL in clinical trials.

## Additional file

Additional file 1: **Figure S1.** Effect of idelalisib on the frozen and thawed samples of ATL patients. **Figure S2.** The apoptotic ATL cells by treatment with idelalisib. **Figure S3.** The viability of non-ATL cells by treatment with idelalisib. **Figure S4.** The apoptotic non-ATL cells by treatment with idelalisib. (DOCX 38 kb)

## Abbreviations

ATL: Adult T-cell leukemia-lymphoma
CCL22: C-C motif chemokine 22
CLL: Chronic lymphocytic leukemia
HTLV-1: Human T-lymphotropic virus type-I
PI3K-δ: Phosphatidylinositol 3-kinase-δ
PTEN: phosphatase and tensin homolog
