# Supplementary material for: Phosphatidylinositol 3-kinase-δ (PI3K-δ) is a potential therapeutic target in adult T-cell leukemia-lymphoma
Source: Biomark Res. 2018 Jul 18;6:24. doi: 10.1186/s40364-018-0138-7 (PMC6052569; doi:10.1186/s40364-018-0138-7)
Supplement: Supplementary file 1 — Figure S1. Effect of idelalisib on the frozen and thawed samples of ATL patients. Figure S2. The apoptotic ATL cells by treatment with idelalisib. Figure S3. The viability of non-ATL cells by treatment with idelalisib. Figure S4. The apoptotic non-ATL cells by treatment with idelalisib. (DOCX 38 kb) [file 40364_2018_138_MOESM1_ESM.docx]

**Additional file 1**

Figure S1.

The frozen and thawed samples of four ATL patients were cultured with 0 - 100µM idelalisib or 3 days. The live and apoptotic cells were quantified in the population of TCR-VβX^+^CD7^–^ ATL cells. Y axis refers to the viability ratio between treated and untreated cells of time-matched samples from the same individuals.

Figure S2.

The apoptotic ATL cells was assayed at day 0 - 10 by flow cytometry. PBMCs were freshly isolated from ATL patients (n=7) and CD8 positive cells were depleted using Dynabeads (Invitrogen). Y axis refers to the ratio of apoptotic cells between treated and untreated cells of time-matched samples from the same individuals.

Figure S3.

The viability of non-ATL cells was assayed at day 0 - 10 by flow cytometry. PBMCs were freshly isolated from ATL patients (n=7) and CD8 positive cells were depleted. The live and apoptotic cells were quantified in the population of TCR-VβX^-^ CD7^+^ non-malignant cells. Y axis refers to the viability ratio between treated and untreated cells of time-matched samples from the same individuals.

Figure S4.

The apoptotic non-ATL cells was assayed at day 0 - 10 by flow cytometry. PBMCs were freshly isolated from ATL patients (n=7) and CD8 positive cells were depleted. Y axis refers to the ratio of apoptotic cells between treated and untreated cells of time-matched samples from the same individuals.

Figure S1.

Figure S2.

Figure S3.

Figure S4.
